# New Insights in Prostate Cancer Development and Tumor Therapy: Modulation of Nuclear Receptors and the Specific Role of Liver X Receptors

**DOI:** 10.3390/ijms19092545

**Published:** 2018-08-28

**Authors:** Laura Bousset, Amandine Rambur, Allan Fouache, Julio Bunay, Laurent Morel, Jean-Marc A. Lobaccaro, Silvère Baron, Amalia Trousson, Cyrille de Joussineau

**Affiliations:** 1 Université Clermont Auvergne, GReD, CNRS UMR 6293, INSERM U1103, 28, place Henri Dunant, BP38, F63001 Clermont-Ferrand, France; laura.bousset@uca.fr (L.B.); amandine.rambur@uca.fr (A.R.); allanfouache76@gmail.com (A.F.); julio.bunay_noboa@uca.fr (J.B.); laurent.morel@uca.fr (L.M.); amalia.trousson@uca.fr (A.T.); cyrille.de_joussineau@uca.fr (C.d.J.); 2 Centre de Recherche en Nutrition Humaine d’Auvergne, 58 Boulevard Montalembert, F-63009 Clermont-Ferrand, France

**Keywords:** prostate cancer, metastasis, LXRs, androgens, estrogens, cholesterol, oxysterols, signaling pathway

## Abstract

Prostate cancer (PCa) incidence has been dramatically increasing these last years in westernized countries. Though localized PCa is usually treated by radical prostatectomy, androgen deprivation therapy is preferred in locally advanced disease in combination with chemotherapy. Unfortunately, PCa goes into a castration-resistant state in the vast majority of the cases, leading to questions about the molecular mechanisms involving the steroids and their respective nuclear receptors in this relapse. Interestingly, liver X receptors (LXRα/NR1H3 and LXRβ/NR1H2) have emerged as new actors in prostate physiology, beyond their historical roles of cholesterol sensors. More importantly LXRs have been proposed to be good pharmacological targets in PCa. This rational has been based on numerous experiments performed in PCa cell lines and genetic animal models pointing out that using selective liver X receptor modulators (SLiMs) could actually be a good complementary therapy in patients with a castration resistant PCa. Hence, this review is focused on the interaction among the androgen receptors (AR/NR3C4), estrogen receptors (ERα/NR3A1 and ERβ/NR3A2), and LXRs in prostate homeostasis and their putative pharmacological modulations in parallel to the patients’ support.

## 1. Introduction

Prostate cancer (PCa) rarely appears before the age of 40 years and is diagnosed in men of approximately 70 years old. Already known risk factors include age, family history, ethnicity, and internal steroid hormones levels, whilst there are also emerging carcinogenic factors, including diet, lifestyle, and exposure to xenobiotics. PCa requires androgens for growth, and androgen deprivation has, for decades, been the principal strategy to treating advanced disease. The increased incidence of PCa in many countries has been partly attributed to changes in diagnostic methods. Despite its controversy, introduction of the prostate-specific antigen (PSA) assay as a screening method during the last decade has led to an increase in incidence. However, this alone does not explain the observed continuing rise. Besides, epidemiological studies have also pointed out that environmental factors could influence PCa risks, even though they are difficult to define [1]. PCa is the paradigm of the endocrine-related tumors, together with breast cancer. Various hormones, mainly steroids and their respective nuclear receptors (NRs), have a prominent role in the development of PCa. Hence, unusual levels of testosterone and dihydrotestosterone (DHT) in prostate tissue are suspected to increase the risk of developing cancer [2], and estrogens are believed to have an important role as well [3,4].

**Figure 1:**
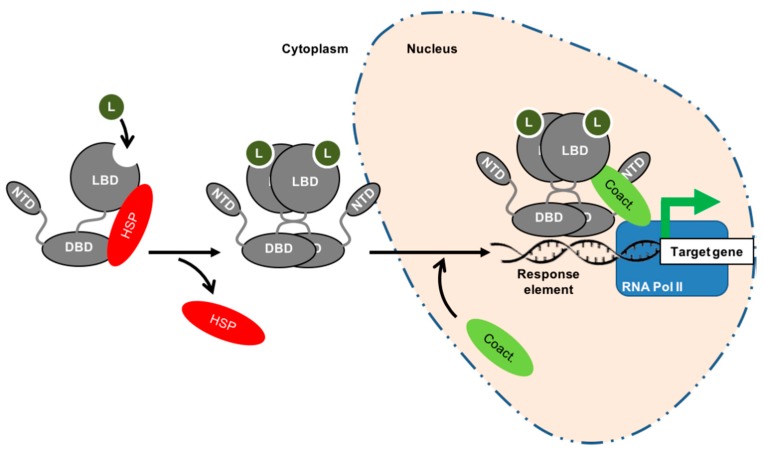
Schematic structure and functioning of steroid receptors. Nuclear receptors are composed of an N-terminal domain (NTD), a DNA-binding domain (DBD) responsible of the binding to the DNA-target sequences usually located within the promoters of the targets genes and a C-terminal ligand-binding domain (LBD), which is specific to the molecule. Canonically, it is admitted that steroid receptors are located within the cytoplasm in the absence of hormone, bound to heat shock proteins (HSP) that impede shuttling to the nucleus. The binding of the steroid (L) allows the chaperones to unbind from the receptor and migrate to the nucleus after a homodimerization. The binding of co-activators (Coact.) makes the recruitment of the transcriptional machinery possible, along with the RNA polymerase II (RNA Pol II), the transcription of the target gene, and the physiological effects.

NRs are part of a superfamily encompassing 48 members within humans. As DNA-binding proteins, they can control the transcription of genes whose products are fundamental for important physiological functions (for a review see Evans and Mangelsdorf [5]). Schematically, NRs are composed of three major independent functioning domains (Figure 1): a N-terminal involved in the regulation of the non-ligand dependent transcriptional activity, a central DNA-binding domain, and a C-terminal ligand-binding domain carrying a potential ligand-binding pocket controlling the ligand-dependent transcriptional activation function [6].

The steroid receptors are within the cytoplasm in the absence of a ligand and are bound to heat shock proteins that impede the shuttling to the nucleus (Figure 1).

**Figure 2:**
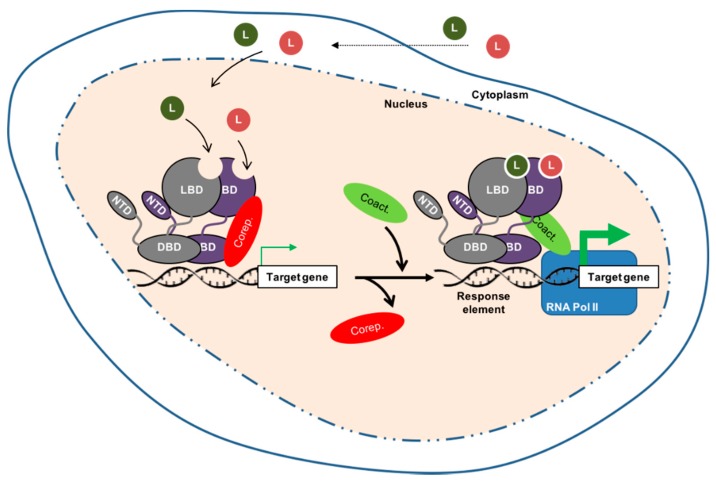
Schematic structure and functioning of nuclear receptors bound as heterodimers with retinoid X receptors (RXR). The non-steroid nuclear receptor (grey) is supposed to be bound with RXRs, a receptor for 9-*cis* retinoic acid (deep purple), to the DNA. In absence of ligand, the transcriptional activity is blocked (thin green arrow) by co-repressors (Corep.). As for the steroid receptors, the binding of co-activators (Coact.) makes possible the recruitment of the transcriptional machinery, along with the RNA polymerase II (RNA Pol II), the transcription of the target gene (thick green arrow), and the physiological effects.

However, other types of NR, such as the liver X receptors (LXRα/NR1H3 and LXRβ/NR1H), function in a heterodimer with retinoid X receptors (RXRs, the receptor for 9-cis retinoic acid, NR2B1-3). In such signaling systems, the heterodimer complex resides bound to DNA in the absence of a ligand, with transcription blocked by the presence of co-repressors (Figure 2).

Ligand binding triggers major conformational changes in the receptor’s ligand-binding domain which leads to the dissociation of chaperones and/or corepressors, nuclear translocation (when necessary) and DNA-binding, and the recruitment of coactivators. Thus, it initiates gene transcription [7] (Figure 1 and Figure 2). Hence, in the presence of an agonist in the ligand-binding pocket, corepressors dissociate and the recruitment of transcriptional coactivators is favored. Reciprocally, interactions with an antagonist represses association with coactivators and enables corepressor recruitment. The ligand-binding domain may also contribute to the modulation of the N-terminal AF-1 through inter-domain crosstalk, allowing activating domains to recruit coactivators individually or in a synergistic manner [7].

## 2. Nuclear Receptors Are Fundamental for Prostate Physiology

Androgen [8] and/or estrogen [9] deregulations have been associated with the development of PCa. Their respective NRs are targeted in the treatment of this tumor. Besides these classical steroid NRs, newly considered steroid receptors such as LXRα and LXRβ [10] have emerged as interesting players in both prostate homeostasis and carcinogenesis [11,12,13]. This section will be focused on the androgen (AR/NR3C4), estrogen (ERα/NR3A1 and ERβ/NR3A2), and oxysterol (LXRs) receptors, and their putative interactions in maintaining prostate physiology.

### 2.1. Role of AR in Prostate Physiology and in PCa

Since pioneering work from Higgins and Hodges was published in 1941 [8], it has been well established that androgen regulation signaling is one of the hallmarks of PCa. If testosterone is the principle circulating androgen, the most active androgen in the prostate remains 5α-dihydrotestosterone (DHT), produced by conversion of testosterone by 5α-reductase type 2 (SRD5A2), which binds their nuclear receptor with two to ten-fold higher affinity than testosterone [14]. DHT exerts its activity through AR-regulated transcription of genes involved in cell differentiation, development, survival, and function of the prostate in adulthood. Androgens may also exert rapid, AR-dependent non-genomic effects. In this process, cytoplasmic AR can induce kinase-signaling pathways such as mitogen-activated protein kinases (MAPK) or phosphoinositide 3-kinase (PI3K)/AKT, which ultimately influence AR signaling through the phosphorylation of AR itself or AR co-regulators (for a review see [15]). Part of this mechanism could explain therapeutic failures when anti-androgens have no effect on androgen target genes despite AR remaining active.

#### 2.1.1. Androgens Are Involved in Prostate Differentiation, Growth, and Functioning

Various knock-out models for AR have pointed out that the initiation of prostate development needs a functional AR [16], even though a correct organization of the future prostate also involves mesenchymal/epithelial interactions, in which AR is required at the stromal level. Cunha and colleagues [17] showed that AR signaling is needed in the mesenchymal compartment for initiation of prostate development, but not in the epithelial compartment. The stromal cells express AR and secrete peptide growth factors in response to androgen signaling [18,19], which contribute to the prostate differentiation. Binding of these growth factors to their respective receptors on basal cells promotes their proliferation and differentiation first into intermediate cells, then into fully differentiated luminal cells [20]. Conversely, basal cells do not have AR [21,22]. In adulthood, maintenance of the prostate gland depends on the balance of growth-survival/apoptosis driven by androgens, which promote the survival of luminal epithelial cells. In animal models, supra-physiological levels of androgens result in an increase in cellular proliferation in the prostate [23]. Conversely, castration induces apoptosis in about 70% of luminal epithelial cells in adult male rats but has little or no effect on basal and stromal cells [24].

#### 2.1.2. Androgens Drive Proliferation of Luminal Epithelial Cells in PCa

The malignant switch from benign luminal cells to cancerous adenocarcinoma cells is poorly understood, but regardless, AR activity still drives proliferation. This applies both in tumors which are dependent on circulating androgens and in more advanced, castrate resistant disease [25,26,27]. This signaling pathway pathologically allows androgen/AR complexes to bind to and enhance expression of survival and proliferation genes that are normally not regulated by these complexes in either intermediate cells or luminal cells [25,26,27]. Using ChIP-on-Chip and ChIP-Seq experiments, research groups have reported thousands of AR direct binding events in PCa cell lines and tissues, furthermore showing different binding patterns depending on the stages of cancer [28,29,30].

### 2.2. Role of ER in Prostate Physiology and in PCa

The physiological functions of estrogens in the prostate, other than their activity as antiandrogens, have been unclear for many years. The development of genetic mouse models and their study have helped decipher the role of 17β-estradiol and its receptors in the prostate. 17β-estradiol activity is mainly mediated through its specific nuclear receptors, ERα (NR3A1) and ERβ (NR3A2) (for a review see Yaşar et al. [31]). As with AR, ERs control transcription via binding to distinct DNA sequences at target genes or through their non-genomic activity. In non-malignant human adult prostate tissue, ERα is mainly expressed in the stromal compartment where it is involved in stromal cell proliferation and differentiation [32], and it can be found in less than 2% of epithelial cells where it drives estrogen-mediation epithelial cell proliferation [33,34]. Conversely ERβ is detected in 90% of epithelial cells [35,36,37], where it promotes cell homeostasis and differentiation [38].

During PCa initiation and progression, stromal ERα expression remains but up to 80% of all epithelial cells show an expression of ERα that rises from 0–2 to 80% in PCa [37]. Inversely, epithelial cells lose ERβ, as it drops from 90 to 15% of cells in PCa [35,39,40]. Finally, the TRAMP and PB-Cre4/*Pten^loxP^* PCa mouse models have helped to understand the respective opposite roles of both ERs, along with the significance of this change that is seen in PCa. While epithelial ERα is thought to be responsible of estrogen-mediated PCa growth, epithelial ERβ is believed to have protective roles [41].

Epidemiological data have shown controversial results regarding the role of circulating estrogens. Local steroid production by prostate tumors, rather than circulating steroids, may play a major role in driving PCa growth in men following androgen deprivation therapy [42]. While current research on the role of intracrine steroids focuses on intratumoral androgens, it is important to note that aromatase (CYP19A1), which catalyzes estradiol production from testosterone, is also altered in PCa tissues [43] and CYP19A1 expression could be 30-fold higher than normal in PCa metastatic tissues [44].

### 2.3. LXRs as Emerging Factors Regulating Prostate Physiology

LXRα and LXRβ, encoded by two distinct genes, are composed of 447 and 460 amino acids, respectively (for a review see [10,45]). They cannot be formally considered as true isoforms; however, they share 77% of their identity within their DNA- and ligand-binding domains. Initially described as orphan receptors, Mangelsdorf’s group demonstrated more than 20 years ago that LXRs were actually the receptors for oxysterols, oxidized derivatives of cholesterol [46,47]. Because oxysterols were described as bona fide ligands, it was suspected that LXRs could be involved in the regulation of cholesterol metabolism. Indeed, this was further demonstrated by the phenotype analysis of LXR-deficient mice [48]. Hence, the activation of both LXRs by their cognate oxysterol ligands reduces the intracellular concentration of cholesterol. This occurs through several means: (1) the increase of the cholesterol efflux via the transcription of *ABCG1* and *ABCA1*, two membrane transporters and target of LXRs; (2) the inhibition of the cholesterol influx by increasing the ubiquitin ligase inducible degrader of the LDL receptor (IDOL), which targets the LDL receptor; (3) the increase of cholesterol metabolism into bile acids (induction of CYP7A1) or steroids (induction of StAR); and (4) the increase of cholesterol storage.

In the prostate, we and others have explored the physiologic role of LXRs. Both isoforms are expressed in the prostate, in epithelial as well as in stromal cells. However, so far, it has not been possible to dissociate the exact role of each LXR isoform as they both compensate each other [49]. Fukuchi et al. first pointed out that ABCA1 was downregulated by AR in LNCaP cells [50]. Furthermore, the same group showed that the activation of LXRs by the synthetic agonist T0901317 slows down LNCaP proliferation [51], suggesting a role of LXRs in the progression of CaP, or at least showing that these receptors could be pharmacologically targeted. LXRs and some of their target genes were found to be less accumulated during the progression of the androgen-dependent into androgen-independent relapsed tumors in a xenograft model [52].

The role of LXRs in the control of the apoptosis of prostate cells was also shown using various natural or synthetic ligands [11]; this occurs through the modulation of the membrane cholesterol content associated with the lipid rafts, thus decreasing the PI3K and survival pathways. Using new cellular models derived from the dorsal prostate, we showed that LXRs control both protein kinase B (or AKT) and MAPK phosphorylation pathways in a normal prostate’s cell cycle [12]. This point is of importance as the Ras/MAPK pathway is essential to maintaining cellular homeostasis because of its implication in cell proliferation and differentiation. Additionally, this pathway is the second major signaling pathway whose deregulation has been associated to prostate tumorigenesis: it has been found up-regulated in 43% of primary tumors and in the greatest majority of metastases [53]. Furthermore, the suppressor of cytokine signaling 3 (SOCS3)*,* which is able to inhibit p42/p44 MAPK signaling [54] and the proliferation and migratory ability of cancer cells, is upregulated upon LXR activation [55,56]. Janus kinase/signal transducers and activators of transcription (JAK/STAT) and Wingless/Integrated (Wnt) pathways could also be deregulated in PCa [53]. Interestingly, LXR activation represses the JAK/STAT signaling pathway in the liver and decreases β-catenin accumulation, a crucial mediator of the Wnt pathway [57].

In parallel, our group pointed out that LXR-deficient mice fed with a high cholesterol diet presented a neoplasia within the prostate epithelium, characterized by the downregulation of the tumor suppressor Homeobox protein NKX3.1 and beta-microseminoprotein MSMB and the upregulation of pro-oncogenic factors such as CyclinD1 and CyclinD2 [58]. This was definitively due to the accumulation of the oncogene and histone methyl transferase enhancer of Zeste Homolog 2 (EZH2), whose overexpression has been described in patients with an aggressive PCa [59]. The question regarding any expression change with cancer progression is unclear. Indeed, Oncomine^®^ analysis did not show any difference for LXRα, while a slight significant difference was observed for LXRβ [58].

**Figure 3:**
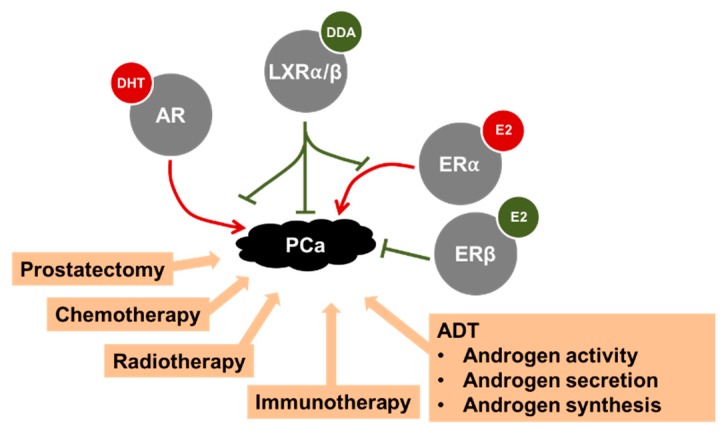
Summary of the various treatments proposed to treat PCa. A focus is made on some nuclear receptors. The androgen (AR) and estrogen receptor (ER) α have deleterious effects on prostate cancer (PCa) progression when activated by their respective ligands dihydrotestosterone (DHT) and 17β-estradiol (E2). Conversely, nuclear oxysterol receptors (LXRα/β) and ERβ block the progression of PCa in animal models when activated by their respective ligands. As indicated, AR and ERα activity in PCa could be modulated by LXRs, directly or indirectly. For more details see the text. DDA: dendrogenin A.

Altogether, ex vivo and in vivo studies suggest that LXRs could be implicated in the progression of PCa and, thus, could represent a pharmacological target for its treatment (Figure 3).

### 2.4. Androgens, Estrogens, and Oxysterols Interact in the Prostate

Despite their specific respective physiological roles on prostate physiology (see above sections), androgens, estrogens, and oxysterols cross interact through the transcriptional activation of their respective NRs.

#### 2.4.1. LXRs Regulate the Availability of Active Steroids

As described above, prostate homeostasis is sensitive to steroids such as androgens and estrogens. All of these molecules derive from cholesterol. Regarding the binding to their respective receptors, the availability of androgens [60] and estrogens can be modulated by sulfotransferases, which inactivate the steroid activity [61]. Previous work has reported that sulfotransferase family 2A member 1 (SULT2A1) deactivating androgens is a LXR-target gene [62]. Indeed, ligand activated LXRs deprive cells from androgens through the increase of SULT2A1. Conversely, LXRs inhibit the expression of steroid sulfatase STS, an enzyme involved in the activation of androgens in prostate [63]. The level of active estrogens is also dependent of the levels of the estrogen sulfotransferase EST/SULT1E1. As for androgens, sulfated estrogens cannot bind to and activate ER activities [64]. 

#### 2.4.2. AR, ER, and LXR Interaction within the Prostate

PC-3 AR-negative cells display a higher accumulation of LXR target genes [65], such as *ABCA1* and *ABCG1* [13,50,52]. A similar accumulation is also seen mouse models and in patients receiving androgen deprivation therapy [66]. Benign prostatic hyperplasia is a pathological situation due to an excessive activity of AR and the production of DHT [67]. Using transgenic animals, we [49] and others [68] described LXRα as a key modulator of the cross talk between the stromal and epithelial compartments, which is essential for the integration of androgen signaling in the prostate and its effect on the epithelium. We pointed out that LXRα-deficient mice have increased secretory activity in the epithelium resulting from a deregulation of the androgen signaling. No clear specific role was identified for LXRs between the epithelial and the stromal compartments, suggesting a complex paracrine network regulated by these receptors [49]. Likewise, Tsui et al. [69] pointed out that LXR expression was higher in androgen-sensitive LNCaP cells than in other PCa cell lines, and activating LXRs by T0901317 in LNCaP cells decreases their AR accumulation and PSA production. Overall, AR and LXRs are definitively interconnected. Even it is not fully demonstrated for the prostate, it has also been shown that ERs down-regulate *LXR*α mRNA in mouse macrophages [70,71], through an estrogen response element in the *LXR*α promoter [70], suggesting a putative interaction between these two classes of receptors.

### 2.5. NR Activity Can Be Altered in Prostate Physiology by Environmental Disrupting Chemicals

Cholesterol-derived molecules are not the only ones that are able to modulate AR, ER, and LXR transcriptional activity (Figure 3). Indeed, environmental disrupting chemicals (EDCs) (reviewed in Delfosse et al. [72]) affect physiology in various ways by mimicking natural endogenous molecule activity, antagonizing their action, or modifying the synthesis, metabolism, and transport of these endogenous compounds. Due to their estrogen-like structure, the main harmful effects of EDCs have been attributed to their interference with hormone signaling mediated by NRs [72]. Hence, the physiologic roles controlled by the targeted NRs are altered. Historically, studies were focused on ERα and ERβ, AR, and thyroid receptors TRα (NR1A1) and TRβ (NR1A2). Then, because disorders of metabolic pathways have been associated to a higher risk of developing PCa, NRs controlling these processes have been the center of interest. Among them, peroxisome proliferator activated receptors (PPARγ/NR1C3), rexinoid receptors (RXRs/NR2B1-3), and LXRα and β/NR1H3 and 2 are abundantly studied due to their potential to be pharmacologically targeted (for a review on LXRs please refer to Maqdasy et al. [10]).

## 3. Lipid Metabolism, Angiogenesis and Immunity Are Altered in PCa

As for other cancers, prostate tumorigenesis is a multifactorial process that depends on cell modifications to allow for proliferation and growth. For that purpose, tumor cells usually (1) adapt their metabolism to produce enough cell components [73] and (2) induce molecular modifications to escape from cell cycle control.

### 3.1. Lipid and Cholesterol Metabolism in Cancer Cells

Among the numerous metabolic alterations that fuel cells and allow for the increased rate of growth and proliferation in cancer cells, the Warburg effect is a metabolic switch where cancer cells reprogram their glucose metabolism for “aerobic glycolysis”, a process usually favored in anaerobic conditions [74]. Another example of an adaptation is citrate and fatty acid production. While citrate, necessary for secretion in prostatic fluid, is produced in large amount, it is used as a substrate for de novo fatty acid synthesis [75], which is found to be increased in PCa development [76].

A deregulation in cholesterol homeostasis has also long been associated to PCa [77,78]. Indeed, White described an “accumulation of crystals of lipid nature in tumors” suggesting that “cholesterol might be associated in some way with the regulation of cell proliferation” [78]. Interestingly this suspicion was later confirmed by Swyer who identified a two-fold increase of cholesterol content when prostates were affected by a hypertrophy [77]. More recently and using imaging data, prostate cancer aggressiveness has been associated with an aberrant accumulation of esterified cholesterol in lipid droplets [79]. However, no clear link has been made between cholesterolemia and Gleason score, positive nodal status, and/or positive surgical margins [80]. Hydroxy-3-methylglutaryl-coenzyme A reductase (HMGCR) is the key enzyme in endogenous de novo cholesterol synthesis and is inhibited by statins. Interestingly, the use of statin by patients suffering a hypercholesterolemia has been associated with improved PCa specific survival, particularly in men undergoing radiotherapy [81].

Additionally, and as presented above, LXRs increase the apoptosis of PCa cell lines by modifying the membrane distribution of cholesterol. Indeed, we pointed out that activated LXRs by various natural or synthetic ligands could induce smaller and thinner lipid rafts and downregulate AKT phosphorylation in these lipid rafts [13]. LXRs also regulate the first step of prostate carcinogenesis since, as previously presented, LXR-deficient mice fed a high cholesterol diet have prostatic intra-epithelial neoplasia while wild-type mice do not. This indicates that LXRs could act as gatekeepers against PCa when cholesterol homeostasis tends to be destabilized.

Conversely, AR and androgens have been implicated in the increase of the intracellular concentration of cholesterol in PCa cells by inducing the accumulation of HMGCoA and transcription factor sterol response element binding protein (SREBP2), which increases cholesterol de novo synthesis, and by decreasing the amount of ABCA1 [82]. AR also decreases LXR activation in PCa by competing for their coactivators. For a review regarding the interaction between AR and LXRs refer to Cariello et al. [83].

Besides an increased endogenous synthesis of lipids, environmental factors such as food intake play a critical role in cancer development. Hence, it has been considered that food intake could represent the principal source of cholesterol [84]. It is thus not surprising that an enriched lipid diet could be described as a risk factor for developing PCa [85]. A similar positive association with a higher risk of developing PCa has been found for the consumption of products of animal origin [86].

### 3.2. Modification of Angiogenesis and Immunity

Immune system dysregulation is another feature of cancer development. Some studies show an interesting correlation between recurrent prostatitis and PCa, suggesting the importance of the immune system in prostate carcinogenesis [87]. Indeed, systemic and in situ inflammation could be observed in the tumor and stromal microenvironment. This inflammation is suspected to contribute to the tumor development by the supply of bioactive molecules, such as growth factors produced by the microenvironment immune cells. PCa has also been associated with the accumulation of inducible nitric oxide synthase (iNOS) [88]. Cyclooxygenase (COX) 2, a pro-inflammatory enzyme synthetizing iNOS is highly expressed in tumor-associated macrophages [89]. In the same way, IL6 is highly expressed in PCa and promotes tumor growth at least in part via PI3K/AKT signaling activation [90,91]. Noteworthy, LXRs downregulate inflammation by the parallel inhibition of iNOS, COX2, and IL6 expression [92,93]. Moreover, LXR activation is able to stimulate an antitumor immune response by promoting the secretion of IFNγ by macrophages and T-cells, a phenomenon that has been associated with an increase in survival of mice injected with lung carcinoma cells [94].

Tumor expansion depends on the availability of nutrients and oxygen and the ability to eliminate the waste produced by the high metabolism of the tumor cells. To avoid the microenvironment becoming hypoxic and poor in nutrients, cancer cells favor angiogenesis by increasing the production of vascular endothelial growth factor (VEGF). In PCa cells, VEGF is highly expressed. Moreover it has been shown that LXRs are able to decrease VEGF signaling by modifying the distribution of cholesterol at the membrane [95,96]. Logically, synthetic LXR agonist T0901317 blocks migration of endothelial cells and vessel tube formation [97].

Altogether, modulation of cholesterol homeostasis has a strong effect both on inflammation and angiogenesis within the prostate, two steps that are important for the progression of PCa to an advanced form of disease. Hence, the fact that identified metabolisms are altered during tumor development and metastasis process allows for the future possibility of using inhibitors of lipid synthesis, inflammation, and/or angiogenesis in patients with PCa.

## 4. Management of Prostate Cancer and Treatments

Even though this review is focused on the NRs in PCa, it is important to have a clear overview of the various management strategies as, according to what was described in the previous sections, anti-steroid therapy or novel molecules targeting LXRs could be applied depending on the stage of PC. Hence, medical care of the patients is based on whether the tumor is localized, if it is advanced, metastasized, or resistant to castration. Apart from classical treatments such as radio- or chemotherapy, specific strategies have been developed to target steroid production and have shown some efficacy. Still, new strategies are to be developed to increase treatment efficacy. The treatments could also be combined in order to increase their efficacy.

### 4.1. Local PCa

Less than 5–10% of patients with low-grade, low-volume tumors will develop a PCa in the 10 years following diagnosis [98]. Based on the usually late age of diagnosis (usually above 70 years of age), deferred treatment and active surveillance are proposed to patients in order to prevent over-treatment that reduces mortality while decreasing the quality of life. About 80% of men with PCa have a localized disease [99]. For localized or locally advanced disease, the choice of treatment will be depending on the risk of progression of the tumor, life expectancy, and patient wishes. According to the European Association of Urology guidelines, radical prostatectomy is usually proposed to patients with low to high-risk PCa since they have a life expectancy >10 years. Radiation therapy is a suitable option for low-risk PCa and should be used in combination with androgen deprivation therapy (ADT) for intermediate/high-risk localized and locally advanced PCa.

### 4.2. Advanced and Metastatic PCa

Median survival for a patient newly diagnosed with metastatic PCa (about 5%) is at least 42 months. However, this is largely heterogeneous [100], and the first line standard approach for patient with advanced PCa is ADT [101]. Three main pharmacological treatments are proposed to block androgen effects. The first is treatment with LHRH ligands [102]. On one hand, agonists (mainly goserelin leuproline, and triptorelin) are able to downregulate the LHRH receptor, resulting in a huge decrease of LH/FSH secretions and testosterone production. On the other hand, LHRH antagonists (abarelix and degarelix) directly block the LHRL receptor and also result in the shut-down of testosterone production. The second treatment is the blockade of androgen synthesis. CYP17, the limiting enzyme for the production of androgens, is upregulated in CRPC [44] and can be inhibited by abiraterone treatment [103]. Ketoconazole has also been shown to block the adrenal steroidogenesis. This metabolic pathway is important as HSD3B1, which converts DHEA to androstenedione in prostate tissue as well as in the adrenal gland, is sometimes found highly expressed in PCa [104]. Likewise, AKR1C3, which converts weak adrenal androgens (e.g., DHEA and androstenedione) into T and DHT, is a good pharmacological target [105]. The third treatment is antiandrogens to antagonize AR and androgen action. These compounds could have either a steroid structure as cyproterone acetate or a non-steroid backbone, such as bicalutamide, flutamide, or nilutamide. Enzalutamide, also a non-steroidal antiandrogen, prevents AR nuclear translocation and has a higher affinity for AR than the widely used bicalutamide [106].

Together with ADT, chemotherapy may be performed. Recent clinical trials and meta-analyses show a 9% improved benefit on overall survival at four years of using docetaxel chemotherapy in combination with ADT for metastatic hormone-sensitive PCa [100].

### 4.3. Castration Resistant Prostate Cancer

Most patients experience tumor growth recovery despite being on ADT within a median of 18 to 24 months [107] and progress to a lethal stage called castration-resistant PCa (CRPC). The emergence of this aggressive form of PCa is diagnosed when blood PSA increases despite a low serum testosterone, followed by a progression of the disease with the appearance of new symptoms and bone or soft tissue lesions [108]. About 33% of men with a rise of PSA level will develop bone metastasis within two years [109], even though patients with CRPC are highly heterogeneous. Various genetic alterations have been associated to this CRPC, such as alterations in PI3K or in Wnt pathways and in the cell cycle or DNA repair processes [110].

However, AR pathway harbors the main alterations. Five general mechanisms are usually described to explain the emergence of CRPC. The first is the overexpression of the protein AR [111,112], even though AR expression is lost in a subset of metastatic CRPC (mCRPC) [113] and the mechanisms associated to this complete androgen independence remain unclear. The second are the mutations of AR [114], which could induce a hypersensitivity to low levels of androgens, an antagonist-to-agonist switch for antiandrogens, or a receptor able to respond to non-canonical agonists like non-androgen steroids. The third is the upregulation of AR coactivators such as ARA70, which plays an important role in the antiandrogen antagonist-to-agonist switch in the DU145 PCa cell line [115], the forkhead box protein (FOXA1) [116], immunoglobulin transcription factor (ITF2), steroid receptor coactivator (SRC1) [117], lysine methyltransferase (EZH2) [118], and aldo-keto-reductase (AKR1C3) [119]. Conversely to breast cancer, no downregulation of co-repressors has been reported so far. The fourth is the activation of AR by tyrosine-kinase receptors linking the androgen regulated pathway with the growth factor signaling pathways. Hence, insulin like growth factor (IGF-1), keratin growth factor (KGF), and epidermal growth factor (EGF) can activate AR in vitro in the absence of a ligand [120]. Likewise, receptor tyrosine-protein kinase erbB-2 (Her-2/neu) overexpression has been observed in 67% of CRPC tissue versus 20% of hormone-naive tissue [121,122] and participates in the activation of AR in absence of androgen through MAPK signaling [123]. Her-2/neu activation also increases AR stability [124]. The firth is the intratumoral synthesis of active androgens. The primary source of androgens for the tumor cells are the testes, even though the adrenal glands [125] could, to a less extent, provide inactive androgens that are transformed into active hormones (5–10%). Additionally, as described above, some tumors synthesize their own active androgens by the abnormal production of cytochrome CYP17A1 and AKR1C3 or a higher activity of SRD5A1/3, which allows for the conversion of testosterone into DHT [126].

### 4.4. New Therapeutic Strategies for PCa Beside Steroids and NRs

For few years, therapeutic strategies have attempted to reduce overtreatment of metastatic CRPC (mCRPC) patients, which only offers little benefits to life expectancy while having a negative effect on quality of life. One strategy to prevent over-diagnosis of PCa has been to limit systematic PSA screening. However, metastatic PCa has been increasing in the USA. As PCa mortality is due to the progression of the disease, the new challenge for physicians and researchers is to propose new options to treat advanced and metastatic forms of PCa.

Chemotherapy with cabazitaxel, a novel taxane-derivate drug, is now a second-line option for docetaxel-resistant cancer. A TROPIC phase III clinical trial demonstrated an overall survival benefit of cabazitaxel (15.1 months) versus mitoxantrone (12.7 months) in docetaxel-resistant CRPC [127].

As PCa preferentially metastasizes in bone (90%) and primarily forms osteoblastic lesions [128], these metastases are incurable and contribute to tumor-specific morbidity and mortality [129]. Hence, radium 223, which is a radiopharmaceutical molecule, similar to calcium, improves overall survival by binding to newly formed bone and emitting radiation to induce apoptosis in the surrounding tumor cells [130]. Bisphosphonates slow down bone resorption and prevent loss of bone mass by binding hydroxyapatite and inhibiting osteoclast activity. Among them, zoledronic acid shows a delays in the emergence of skeletal-related events by 36% [131]. Targeting the bone microenvironment can also reduce skeletal-related events. Denosumab is a monoclonal antibody, which binds to receptor activator of nuclear factor kappa-B (RANK) ligand, interfering with the activation of RANK at the surface of osteoclasts and thus inhibiting their proliferation, function, and survival while decreasing bone resorption. This molecule delays the time of apparition of first bone metastasis for about four months [132].

Cancer therapy is now facing the new era of immunotherapy. Recently, focus was made on immunotherapy of PCa since sipuleucel-T demonstrated significant, even limited, benefit on overall survival in a clinical trial on asymptomatic or little symptomatic mCRPC patients [133].

Even in CRPC, AR remains active and PCa cells are sensitive to a second-line of ADT. For CRPC patients, specific drugs have been developed such as abiraterone acetate and enzalutamide (see above).

Unfortunately, initial responders to second generation ADT inevitably become resistant to enzalutamide and abiraterone, and others develop acquired resistance [134]. So, for the last few years, research has been focused on the comprehension of the molecular mechanisms inducing these resistances [134].

The discovery of new mechanisms of resistance will allow for the deciphering of new molecular pathways and encourage the development of future molecules, such as the currently under trial apalutamide (ARN-509, Janssen, Horsham, PA, USA), darolutamide (ODM-201, Orion, Espoo, Finland), proxalutamide (GT0918, Suzhou Kintor Pharmaceuticals, Suzhou, China), seviteronel (Viamet Pharmaceuticals, Research Triangle Park, NC/Innocrin Pharmaceuticals, Durham, NC, USA), ASN001 (Asana Biosciences, Lawrenceville, NJ, USA), and TRC253 (Janssen).

Recent results suggest that oxysterols and LXRs have a protective role against progression and dissemination of tumor cells. Indeed 27-hydroxycholesterol, a LXR ligand, reduces the invasive potential of LNCaP and PC3 PCa cells in in vitro cell invasion assays [135]. Pencheva and colleagues demonstrated in a melanoma model that treatment with an LXR agonist affected secretion of APOE by stromal cells resulted in a decrease in tumor growth, neoangiogenesis, and metastatic dissemination [136]. Likewise, Segala et al. pointed out that dendrogenin A, a newly discovered cholesterol metabolite and activator of LXRs, is able to specifically induce lethal autophagy of cancer cells in vitro and in vivo [137].

## 5. Conclusions

As already presented, cholesterol is an important molecule whose concentration needs to be controlled in prostate physiology. Since cholesterol is associated with the aggressiveness of prostate adenocarcinoma, targeting its metabolism appears to be an interesting theoretical therapy. Two pathways can be targeted: the mevalonate pathway, which is inhibited by the statins, and the LXR signaling pathway, which could be activated to tighten the control of cholesterol homeostasis. The modulation of both pathways will have a hypocholesterolemic effect.

Various epidemiological studies and meta-analyses have shown that the use of statins reduces the risk of PCa mortality [138] and, more importantly, decreases the risks associated with the development of advanced PCa [139] to present aggressive pathological features in histological analysis of prostatectomy specimens [140] or to develop distant metastasis in patients who were diagnosed with non-metastatic PCa [141]. A decrease in the time of response to ADT has also been reported in patients with hypercholesterolemia. Conversely, statins delay the emergence of CRPC in ADT treated patients [142]. However, it should be kept in mind that statins have numerous side effects.

Since the primary use of statins in PCa therapy is still controversial, the development of new selective liver X receptor modulators (SLiMs, [143]) is theoretically a good option to regulate the intra-prostatic levels of cholesterol. These tissue-specific LXR agonists would modulate intracellular levels of cholesterol in prostate tissue, be specific for each LXR isoform, and prevent any highly adverse side effect. Such a specific SLiM, GW6340, has already been developed. It promotes macrophage reverse cholesterol transport in vivo and thus exerts an anti-atherogenic effect without side effects linked to hepatic LXRs activation like elevated plasma triglycerides [144]. As LXRα seems to be the isoform responsible for the hepatic adverse effects observed upon pan-LXRs activation, the development of LXRβ isoform-specific agonists actually mobilizes intense efforts from researchers. Currently, many molecules are under development [145]. Recently, BMS-852927, a novel partial LXRβ-selective compound, was successfully tested in healthy human volunteers in a phase 1 ascending-dose study [146]. This study reported an increase in plasma and hepatic lipids and a decrease in circulating neutrophils in mice that was not observed in monkeys. These findings highlight the greatest difficulty in drug discovery to predict clinical responses in animal models. To date, RGX-104, a LXRβ-selective agonist, is currently being tested in patients with advanced solid malignancies and lymphoma (ClinicalTrials.gov Identifier: NCT02922764). Expected effects of this molecule are a stimulation of antitumor immunity and an inhibitory action on angiogenesis. Likewise, Segala et al. showed that dendrogenin A is a natural specific activator of LXR usable in the treatment of cancers [137].

As the development of selective LXR agonists and in vitro/in vivo test is ongoing, the safe and effective use of SLiMs appears to will be promising notably for prostate cancer treatment, as well as other steroid-dependent tumors. Given the heterogeneity of the mechanisms of emergence of castration-resistance, the focus of interest should be combinatorial treatment in cancer therapeutic. SLiMs may be used in combination with current standard of care for the treatment of advanced PCa/mCRPC, like ADT, chemotherapy, or immunotherapy to potentiate their effects or to target multiple cancer-associated pathways and to reduce the risk of resistance development overall. Another benefit of combinatorial therapy is to diminish doses of administrated drugs and, likewise, to reduce deleterious side effect. In a xenograft mouse model of melanoma, combining LXR agonist treatment to frontline chemotherapy (dacarbazine) has shown a synergistic effect in reducing tumor growth [136]. This seems to be a direction for the future.

## Abbreviations

ABCA1/G1	ATP-binding cassette A1/G1
ADT	androgen deprivation therapy
AF1/2	activating function 1/2
AR	androgen receptor
COX2	cyclooxygenase 2
DHT	dihydrotestosterone
CRPC	castration-resistant prostate cancer
mCRPC	metastatic CRPC
ER	estrogen receptor
EST	estrogen sulfotransferase
EZH2	enhancer of Zeste homolog 2
Her-2/neu	receptor tyrosine-protein kinase erbB-2
HMGCR	hydroxy-3-methylglutaryl-coenzyme A reductase
IDOL	inducible degrader of the LDL receptor
iNOS	inducible nitric oxide synthase
JAK/STAT	Janus kinase/signal transducers and activators of transcription
LDLR	LDL receptor
LXR	liver X receptor
MAPK	mitogen-activated protein kinases
MSMB	beta-microseminoprotein
NKX3.1	homeobox protein Nkx-3.1
NR	nuclear receptor
PCa	prostate cancer
PI3K	phosphoinositide 3-kinase
PSA	prostate specific antigen
RXR	retinoid X receptor
SOCS3	suppressor of cytokine signaling 3
SR5A2	5α-reductase type 2
SREBP	sterol response element binding protein
SLiMs	selective liver X receptor modulators
SULT2A1	sulfotransferase family 2A member 1
VEGF	vascular endothelial growth factor
Wnt	Wingless/Integrated
